# Non-image-forming vision as measured through ipRGC-mediated pupil constriction is not modulated by covert visual attention

**DOI:** 10.1093/cercor/bhae107

**Published:** 2024-03-23

**Authors:** Ana Vilotijević, Sebastiaan Mathôt

**Affiliations:** Department of Psychology, University of Groningen, Grote Kruisstraat 2/1 9712TS Groningen, The Netherlands; Department of Psychology, University of Groningen, Grote Kruisstraat 2/1 9712TS Groningen, The Netherlands

**Keywords:** pupil, visual attention, iprgcs, non-image-forming pathway

## Abstract

In brightness, the pupil constricts, while in darkness, the pupil dilates; this is known as the pupillary light response (PLR). The PLR is driven by all photoreceptors: rods and cones, which contribute to image-forming vision, and intrinsically photosensitive retinal ganglion cells (ipRGCs), which mainly contribute to non-image-forming vision. Rods and cones cause immediate pupil constriction upon light exposure, whereas ipRGCs cause sustained constriction throughout light exposure. Recent studies have shown that covert attention modulated the initial PLR; however, it remains unclear whether the same holds for the sustained PLR. We tested this by leveraging ipRGCs’ responsiveness to blue light, causing the most prominent sustained constriction. While replicating previous studies by showing that pupils constricted more when either directly looking at, or covertly attending to, bright as compared to dim stimuli (with the same color), we also found that the pupil constricted more when directly looking at blue as compared to red stimuli (with the same luminosity). Crucially, however, in two high-powered studies (*n* = 60), we did *not* find any pupil-size difference when covertly attending to blue as compared to red stimuli. This suggests that ipRGC-mediated pupil constriction, and possibly non-image-forming vision more generally, is not modulated by covert attention.

## Introduction

The pupillary light response (PLR) refers to the constriction of the pupil when exposed to light and the dilation of the pupil when exposed to darkness. The PLR is driven by all photoreceptors: rods, cones, and intrinsically photosensitive retinal ganglion cells (ipRGCs) (McDougal and Gamlin 2015; Mathôt 2018; Barrionuevo et al. 2023). Rods and cones play a pivotal role in image-forming vision by contributing to the perception of visual details, colors, and shapes (Schmidt and Kofuji 2008). In contrast, ipRGCs play a crucial role in non-image-forming vision, that is, visual functions that occur without conscious visual awareness (nonhuman studies:Berson 2003; human studies: Mathôt 2018; Zele et al. 2011; Provencio et al. 2000; Barrionuevo et al. 2023; reviews: Schmidt and Kofuji 2008; McDougal and Gamlin 2015).

The contribution of rods and cones, and thus of the image-forming pathway, to the PLR is mainly to the initial phase of pupil constriction that is triggered about 200 ms after the onset of a stimulus (Gamlin et al. 2007; McDougal and Gamlin 2010; Chakraborty et al. 2022; Gamlin 2022). However, the input from cones and (although perhaps to a lesser extent; McDougal and Gamlin 2010) rods starts to decrease after around 1.5 to 2 s, at which point the photoreceptors start to adapt. The contribution of ipRGCs, and thus of the non-image-forming pathway, to the PLR is mainly to the sustained phase of pupil constriction that emerges about 5 to 10 s after the onset of a stimulus, around the time that rods and cones start to be heavily adapted (McDougal and Gamlin 2010). ipRGCs receive input from both rods and cones, but they also possess their own photopigment, called melanopsin, which allows them to respond even without input from rods and cones (Berson et al. 2002; Dacey et al. 2005; Gamlin et al. 2007; Markwell et al. 2010; Mathôt 2018; Gnyawali et al. 2022; Barrionuevo et al. 2023). ipRGCs are mostly sensitive to blue light because the peak sensitivity of the photopigment melanopsin is around 482 nm (Berson 2003; Adhikari et al. 2016; Mathôt 2018). Thus far, ipRGCs have been found to play an important role in regulation of the circadian rhythm: when ipRGCs are exposed to light, particularly in the blue spectrum, they send signals to the suprachiasmatic nucleus (SCN) in the brain, which is considered the central pacemaker of the circadian system (Zele et al. 2011). Interestingly, although their main contribution is to non-image-forming vision, some studies have also found that ipRGC stimulation modulates V1 activity and affects perceptual experience in humans (Spitschan et al. 2017; Allen et al. 2019). Importantly, ipRGCs are much less prone to adaptation than rods and cones are (Wong 2012). In sum, although consistent pupil constriction to a long-lasting stimulus, such as daylight, seems to be a unified response, it actually consists of two components: an initial phase, driven by (image-forming) rods and cones, and a sustained phase, driven mainly by (predominantly non-image-forming; but see Spitschan et al. 2017; Zele et al. 2018) ipRGCs.

Previous studies have shown that the PLR is susceptible to covert attention (Binda et al. 2013; Mathôt et al. 2013; Naber et al. 2013; Unsworth and Robison 2017). In a study by Mathôt et al. (2013), participants were presented with a display that was vertically divided into a bright and a dark half. At the same time, participants were cued toward either the bright or the dark side, which predicted the location of an upcoming target. The results showed that the PLR is modulated by covert visual attention: the pupil constricted when covertly attending to the bright side as compared to the dark side, even when visual input and gaze position was constant. Importantly, this study (as well as other studies on the same research question) focused on measuring only the first 2 s after the cue presentation. This time window captures only the initial phase of the PLR, which is driven by rods and cones. Therefore, this study and others leave open the question of whether the sustained phase of the PLR, which is driven by ipRGCs (and thus by non-image-forming vision), is also susceptible to covert attention.

We addressed this open question in the present study. To do so, we created a paradigm that rests on two assumptions: (i) The initial pupil constriction, which refers to the smallest pupil size observed approximately 500 ms after stimulus onset, is driven by rods and cones and *not* by ipRGCs. This has been shown by (Gamlin et al. 2007; Gamlin 2022; but see also Table 1 in McDougal and Gamlin 2010), who compared the pupil responses of normal macaque monkeys to those whose rod and cone contributions were pharmacologically blocked. The results showed that the onset of pupil constriction in macaque monkeys with blocked rods and cones was delayed and reduced, whereas the sustained pupil response remained intact (Fig. 3A and B in Gamlin 2022). In other words, ipRGCs are *not* involved in the initial pupil response. This is crucial because it implies that if the initial pupil constriction to two different stimuli is equated, then any differences in pupil size that arise later reflect ipRGC contribution; (ii) ipRGCs are predominantly responsive to blue light, thus causing the most prominent sustained constriction in response to the blue light. This property of ipRGCs reflects the peak sensitivity of their photopigment melanopsin (Berson 2003; Adhikari et al. 2016; Mathôt 2018). Our paradigm capitalizes on this by using blue stimuli to activate ipRGCs and using red stimuli as control stimuli that activate ipRGCs less strongly.

Building on the aforementioned assumptions, we first conducted a calibration experiment in which we established intensities of red and blue that resulted in an equally strong initial pupil constriction. We then conducted a validation experiment that involved directly looking at calibrated red or blue displays for a duration of 15 s. If our assumptions hold and our calibration procedure has worked, then we should observe two things: (i) No variation in the initial pupil response when exposed to isoluminant red and blue displays because the initial pupil response is driven only by rods and cones, and we have ensured that their input is equated. (ii) A greater constriction in the sustained pupil response when exposed to blue displays as compared to red displays, because the sustained pupil response is primarily driven by ipRGCs, which are maximally responsive to blue light. This is exactly what we observed (Fig. 2, upper row, right column).

**Fig. 1:**
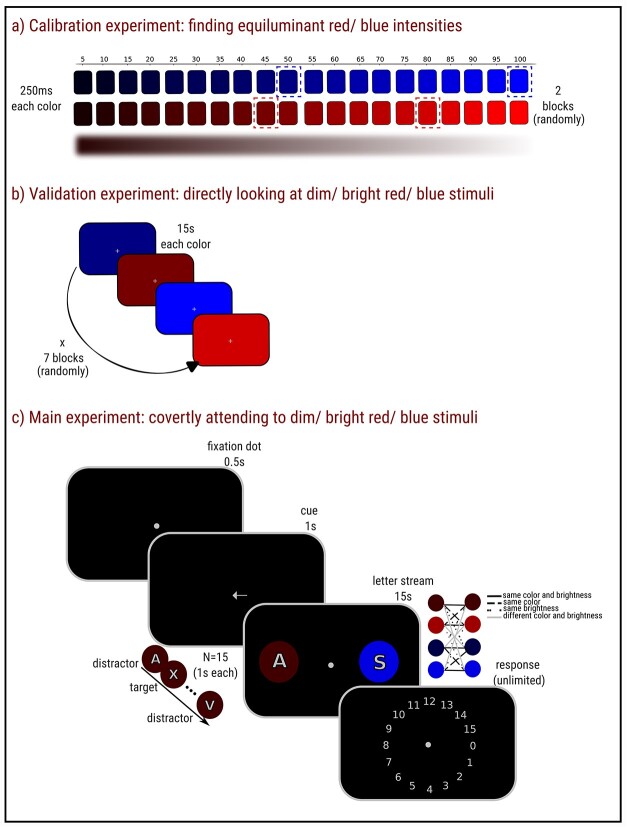
The experimental procedure. a) The calibration experiment. Participants passively looked at the center of the screen while different intensities of red and blue colors were presented in the background. Each color intensity, ranging from 0 to 100 in steps of 5, was presented twice in a random order. Each color intensity was shown for 250 ms followed by a black screen (3,000 ms). The outcome of the calibration experiment were participant-specific isoluminant intensities of dim/bright red and blue. The average color intensities are highlighted by dashed rectangles for illustrative purposes. b) The validation experiment. The color intensities that were obtained in the calibration experiment were now used in the validation experiment (and later in the main experiment). Here, only those four color intensities were presented for 15 s. Each color intensity was presented seven times in a random order. c) The main experiment. Each trial began with a presentation of a fixation dot for 0.5 s, that was followed by a cue presentation lasting for 1 s. The cue indicated the side to which participants should covertly attend. Finally, two streams of letters appeared on both sides of the screen. Both streams consisted of 15 letters. Only the to-be-attended stream contained the targets (“X” letters). The letters on each side were superimposed on placeholder-filled circles that were either dim/bright red/blue. Participants covertly attended to the indicated stream (to-be-attended stream) and reported the number of targets.

We then proceeded to examine whether this effect is susceptible to covert attention in the main experiment. We designed a task in which participants covertly attended to one of two streams of letters. Crucially, we manipulated the brightness (dim/bright) and the color (red/blue) of the placeholders on which the letters were superimposed while measuring participants’ pupil sizes (Fig. 1c). We predicted that pupil size should be smaller when attending to bright stimuli as compared to dark stimuli (given a constant color), and that this should be the most prominent in the initial pupil response, implying that rod-and-cone-mediated pupil constriction, and likely image-forming vision more generally, is modulated by covert visual attention; this prediction is firmly based on previous studies (Binda et al. 2013; Mathôt et al. 2013; Naber et al. 2013; Unsworth and Robison 2017) Crucially, we also tested whether pupil size would be smaller when attending to blue stimuli as compared to red stimuli (given a constant brightness); if so, this should be reflected only in the sustained pupil response and imply that ipRGC-mediated pupil constriction is modulated by visual attention. If not, this would imply that ipRGC-mediated pupil constriction, and likely non-image-forming vision more generally, is *not* modulated by visual attention.

## Materials and methods

All experimental methods and analyses were preregistered at https://osf.io/pva97/.

### Participants

In total, 91 participants, psychology students from the University of Groningen, were recruited and participated in the calibration and validation experiments. From these, 30 participants (predetermined sample size) were eligible for participation in the main experiment upon signing the informed consent form. All participants had normal or corrected-to-normal vision. Participants received course credits for their participation. On the basis of a checklist developed by the Ethical committee (EC-BSS) at the University of Groningen, the study was exempt from full ethical review (PSY-2223-S-0165).

### Apparatus and data acquisition

The experiments were programmed in OpenSesame (Mathôt et al. 2012) using PyGaze for eye tracking (Dalmaijer et al. 2014). Stimuli were presented on a 27-inch PROLITE G2773HBS-GB1 (EOL) monitor (1,920 × 1,080 pixels resolution; refresh rate: 60 Hz; maximum output: 300 cd/m^2^ typical) and an EyeLink 1000 (sampling frequency of 1,000 Hz; SR Research), was used for eye tracking and pupillometry. Participants’ right eyes were recorded. All pupil size recordings were direct. All experiments were conducted in a dimly lit room (lab illuminance: 39 lux).

### Experimental design and procedure

The experimental procedure consisted of three parts: the calibration, the validation, and the main experiment. The first two parts lasted for about 20 min and served as an entry point to the main experiment (see below). An essential aspect of the main experiment involved orthogonally manipulating the brightness (dim/bright) and the color (red/blue) of letters’ placeholders to which participants covertly attended. To ensure that different colors of the same brightness (dim red—dim blue or bright red—bright blue) did in fact not differ in brightness, the calibration and validation experiments were participant-specific. That is, the calibration experiment aimed to determine participant-specific isoluminant intensities of dim/bright red and blue that were subsequently validated in the validation experiment and later (for eligible participants) used in the main experiment. By doing so, we ruled out the possibility that any pupil-size differences observed when presenting colors within the same brightness category were due to variations in luminance between red and blue but rather were due to differential activation of ipRGCs.

The calibration and validation experiments were conducted on the same day, whereas the main experiment occurred within a range of 1 to 7 days after. Prior to the start of each part, after making sure that the participant was well seated at about 60 cm distance from the computer, an eye-tracking calibration–validation procedure was run. A chin rest was used to keep the participant's head in a stable position. In addition to the eye-tracking calibration–validation procedure, a 1-point eye-tracker recalibration (“drift-correction”) was performed before each trial.

### Calibration experiment: finding isoluminant red/blue intensities

The goal of the calibration experiment was to find isoluminant intensities of red and blue. Specifically, we used an automated procedure to determine participant-specific intensities of red and blue that induced the same initial pupil constriction, based on the assumption that this initial constriction is driven only by rods and cones and not by ipRGCs (for similar calibration procedures, see Kinzuka et al. 2022; Mathot et al. 2023; Wardhani et al. 2022). During the calibration experiment, participants looked passively at a central fixation cross while they were exposed to dim/bright red/blue colors presented across the entire display for 250 ms followed by 3,000 ms of a black screen (Fig. 1a). The stimulus’ intensities varied from 0% to 100% (of the monitor’s maximum brightness) in steps of 5%. Each color intensity was presented twice, and the colors were presented in a random order.

For each display presentation, we determined the strength of pupil constriction by fitting a pupil-constriction template (based on the pupil constriction of the last author) to the pupil response. The fitting procedure had four parameters: a horizontal shift, reflecting the latency of the response; a vertical shift, reflecting baseline pupil size; a horizontal scaling, reflecting the maximum constriction velocity; and a vertical scaling, reflecting the constriction strength. For our purpose, constriction strength was the relevant parameter.

Next, we fitted a second-order polynomial to predict constriction strength from stimulus intensity, separately for red and blue displays. We then determined a pair of red and blue intensities that resulted in the strongest constriction that could be matched, such as 100% blue and 83% red or 89% blue and 100% red (one of the colors was always 100%). These intensities were used as the bright intensities. Next, we used the 50% blue intensity and the red intensity that matched in terms of constriction strength as the dim intensities, such as 50% and 45% red (blue was always 50%). The final outcome of the calibration experiment were two (bright, dim) isoluminant intensities for both red and blue.

Participants were eligible for participation in the main experiment only if the calibration experiment revealed a systematic and biologically plausible pattern of pupil constriction, based on visual inspection of the data. The average color intensities of eligible participants were as follows: For dim blue, the intensity was 49% (luminance: 2.04 cd/m^2^; illuminance: 3 lux; HEX code: #00007c; CIE coordinates: *x* ≈ 0, *y* ≈ 0); for bright blue, the intensity was 100% (luminance: 10.60 cd/m^2^; illuminance: 20 lux; HEX code: #0000fe; CIE coordinates: *x* ≈ 0, *y* ≈ 0); for dim red, the intensity was 45% (luminance: 4.36 cd/m^2^; illuminance: 0 lux; HEX code: #720000; CIE coordinates: *x* ≈ 1, *y* ≈ 0); and for bright red, the intensity was 80% (luminance: 19.34 cd/m^2^; illuminance: 8 lux; HEX code: #cc0000; CIE coordinates: *x* ≈ 1, *y* ≈ 0) (see Fig. 1a highlighted colors).

### Validation experiment: directly looking at dim/bright red/blue stimuli

Once these intensities were obtained, the validation experiment started, in which participants were exposed to a prolonged presentation (15 s) of only the obtained participant-specific intensities of each color, followed by 2,500 ms of the black screen, while participants passively fixated the center of the screen (Fig. 1b). Each color was presented seven times, resulting in 28 trials (4 × 7) in total. The validation results served as another entry criterion to the main experiment: only participants who, based on visual inspection of the data, showed a systematic and biologically plausible pattern of gradually emerging stronger constriction during the presentation of the blue color both under dim and bright light conditions during direct looking were invited to participate in the main experiment. Since the decision was made on a level of a single participant (*n* = 1), no statistical tests were performed and this was decided based on visual inspection.

As described under Participants, only a minority of participants (30 of 91) was invited to participate in the main experiment. This is because perfect calibration is difficult to achieve (e.g. due to blinking or recording artifacts) and yet the quality of the calibration is crucial for the validity of the main experiment; we therefore chose to include only participants for whom we were sure that the quality of the calibration was very high. Participants who did not meet the eligibility criteria often nevertheless had stronger pupil constriction when exposed to blue as compared to red light. However, this increased response was typically observed only under one lighting condition, mainly in bright light. To be eligible for our study, participants needed to show pupil constriction under both dim and bright conditions. Noneligible participants never took part in the main experiment.

### Main experiment: covertly attending to dim/bright-red/blue stimuli

Each trial began with a presentation of a fixation dot (0.29°) for 0.5 s, followed by a cue (1.16°) for 1 s. The cue was an arrow pointing either left or right, and indicating the side to which participants should covertly attend. The cue was 100% valid. Next, two streams of letters (gray letters with a black outline; HEX code: #5e5c64; CIE coordinates: *x* ≈ 0.37, *y* ≈ 0.36; 2.31°) appeared on both sides of the screen (one-side eccentricity: 13.87° in total). The letters on each side were superimposed on placeholders, which were filled circles (2.89°) that were either dim/bright red/blue (Fig. 1c). The exact intensities of color–brightness combinations were derived from the calibration experiment and were participant-specific (the average intensities are highlighted in Fig. 1a for illustrative purposes). Both streams (to-be-attended stream and to-be-ignored stream) displayed 15 letters in total, with a pace of 1 s per letter. The streams were not the same, and only the to-be-attended stream contained targets (the letter “X”). The color and brightness of the placeholder circles of the to-be-attended and to-be-ignored stream were fully crossed, featuring 16 combinations [4 (*dim red*/*dim blue*/*bright red*/*bright blue*) × 4] in total. The side of the to-be-attended stream was randomly varied. Participants’ task was to covertly attend to the cued stream, count the number of targets that appeared, and report this number at the end of each trial. Each letter had a 20% probability of being a target, resulting in an unpredictable number of targets and a constant hazard rate to encourage sustained attention (i.e. the chance of the next letter being a target was always the same). The experiment consisted of 6 practice (not analyzed) trials and 224 experimental trials. Experimental trials were shuffled and divided in seven blocks.

### Data preprocessing

Following the workflow for preprocessing pupillary data that we described elsewhere (Mathôt and Vilotijević 2022), we first interpolated blinks and downsampled the data by a factor of 10. Next, we converted pupil size measurements from arbitrary units to millimeters of diameter by using the formula specific to our lab (Wilschut and Mathôt 2022). Finally, we baseline-corrected the data by subtracting the mean pupil size during the first 50 ms after the onset of the Letter stream (baseline period) from all subsequent pupil-size measurements on a trial-by-trial basis.

### Data exclusion

First, we checked whether participants made eye movements and excluded trials in which the deviation from the center of the screen was larger than 6.93° (halfway to the letter stream) and lasted longer than 10 ms (681 trials excluded). Trials containing baseline pupil sizes of ±2 *z*-scores were considered outliers, and hence excluded from the data (442 trials excluded). In total, 1,123 trials (16.71%) were excluded from the data.

## Results

### Validation: directly looking at dim/bright red/blue stimuli

First, we used the results from the validation experiment to analyze pupil size changes when participants were directly looking at dim/bright red/blue. We ran two linear mixed-effects (LME) analyses to investigate the effect of brightness (dim vs. bright) and, separately, the effect of color (red vs. blue) on the pupil. We used the entire 15 s trace as a window of interest. For the analysis that looked into the effect of brightness on the pupil, we aggregated data across colors, so that both dim red and dim blue trials were coded as “dim” and contrasted to both bright red and bright blue trials, which were coded as “bright.” Our model included mean pupil size as a dependent variable, brightness (dim vs. bright) as a fixed effect, and by-participant random intercepts and slopes. Similarly, for the analysis that looked into the effect of color on the pupil, we aggregated data across brightness, so that both dim red and bright red trials were coded as “red” and contrasted to both dim blue and bright blue trials, which were coded as “blue.” Our model included mean pupil size as a dependent variable, color (red vs. blue) as a fixed effect, and by-participant random intercepts and slopes.

**Fig. 2:**
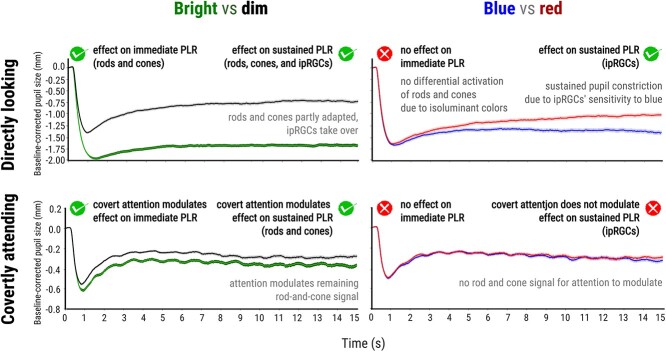
The pupillary results during direct gaze (the validation experiment; upper row) and covert attention (the main experiment; lower row). Pupil size when directly looking and covertly attending to bright vs. dim (left column) and blue vs. red (right column). *Note:* Error bars represent the standard error.

We found a main effect of brightness (*b* = 0.84, *SE* = 0.03, *t* = 29.27, *P* < 0.001), reflecting that the pupil constricted more when directly looking at bright as compared to the dim displays. We also found a main effect of color (*b* = 0.19, *SE* = 0.04, *t* = 4.63, *P* < 0.001), reflecting that the pupil constricted more when directly looking at the blue trials as compared to the red trials (Fig. 2 upper row).

Qualitatively, we also observed the typical pattern of an initial equivalent constriction to blue and red, which reflects that we had successfully matched the intensities of red and blue, followed by a gradually emerging difference such that constriction is more sustained when looking at a blue as compared to a red display. This pattern is the behavioral hallmark of the involvement of ipRGCs (Mathôt 2018; Barrionuevo et al. 2023).

### Main experiment: covertly attending to dim/bright red/blue stimuli

Next, we used the results from the main experiment to analyze pupil size changes when participants were covertly attending to dim/bright red/blue. To test this, we used the python library time_series_test, which combines cross-validation with LME modeling in a way that is suitable for pupil-size data (Mathôt and Vilotijević 2022) (Since this analysis is very time-consuming, we downsampled the data once again by a factor of 10.). The idea behind it is that the cross-validation localizes the sample at which the effect is strongest within the full letter stream time window and then conducts a single LME analysis on these samples. We ran this analysis two times to investigate the effect of brightness (bright vs. dim) and the effect of color (red vs. blue) on the pupil. For the analysis that looked into the effect of brightness on the pupil, we selected only the trials in which the to-be-attended and to-be-ignored streams’ placeholder had the same color (same color trials; see Fig. 1c). Next, we aggregated data across colors, so that both dim red and dim blue trials were coded as “dim” and contrasted to both bright red and bright blue trials, which were coded as “bright.” Our model included mean pupil size as a dependent variable, brightness (dim vs. bright) as a fixed effect, and by-participant random intercepts and slopes. We used the entire 15 s trace as a window of interest. Similarly, for the analysis that looked into the effect of color on the pupil, we selected only the trials in which to-be-attended and to-be-ignored streams’ placeholder had the same level of brightness (same brightness trials; see Fig. 1c). Next, we aggregated data across brightness, so that both dim red and bright red trials were coded as “red” and contrasted to both dim blue and bright blue trials, which were coded as “blue.” Our model included Mean pupil size as dependent variable, Color (red vs. blue) as a fixed effect, and by-participant random intercepts and slopes.

We found a main effect of brightness (*z* = 5.11, *P* < 0.001, tested at samples 170, 490), suggesting that the pupil constricted more when covertly attending to bright as compared to dim placeholders. However, we did not find a main effect of color, (*z* = 0.32, *P* = 0.746, tested at samples 1,160, 1,610, 1,600, and 1,500), suggesting that there were no differences in pupil constriction when covertly attending to blue as compared to red placeholders (Fig. 2 lower row) (for more detailed differences, see Appendix S1).

**Fig. 3:**
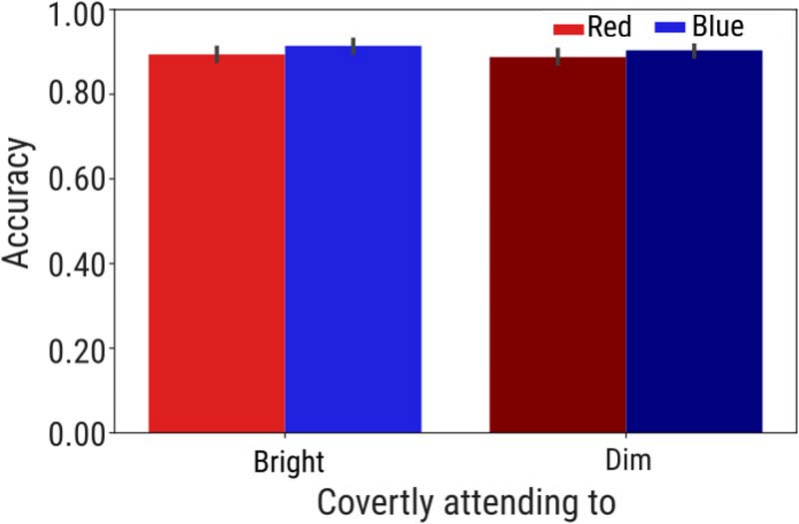
The behavioral results. Accuracy as a function of color and brightness of the covertly attended stream. *Note:* Error bars represent the standard error.

To rule out the effect of task difficulty on pupil size, we checked the accuracy across different conditions. We ran a generalized LME analysis, including accuracy as a dependent variable, color (blue vs. red), brightness (bright vs. dim) of the covertly attended stream, and their interactions as a fixed effects and by-participant random intercepts and slopes for each fixed effect. We found no main effects (color: *b* = −0.25, *SE* = 0.16, *t* = −1.54, *P* = 0.123; brightness: *b* = −0.15, *SE* = 0.15, *t* = −0.97, *P* = 0.329), nor the interaction (*b* = 0.02, *SE* = 0.16, *t* = 0.18, *P* = 0.135), suggesting that the accuracy did not differ across different conditions (Fig. 3).

### Main experiment: covertly attending to bright red/blue (exploratory analysis)

We also checked the effect of color at the different levels of brightness. While there appeared to be a small effect of color under the bright condition, this did not reach statistical significance upon testing (*z* = −0.05, *P* = 0.962, tested across samples 1,450, 50, and 1,610). However, our analysis encompassed the entire 15 s trace, and one of the tested time points was just 500 ms after stimulus onset, suggesting that the cross-validation procedure had selected an unlikely time point (which sometimes happens in noisy data). To address this, we refined our approach by focusing solely on the last three seconds of the stream; this now revealed a significant main effect of color (*z* = 2.43, *P* = 0.015, tested at samples 1,460, 1,400, and 1,450). This is a post-hoc and weak result in a subset of the data (840 out of 6,720 trials), but nevertheless it raises the question of whether pupil size might be smaller when covertly attending to blue compared to red if the colors are sufficiently bright. Therefore, we conducted a preregistered follow-up study in which we focused only on bright colors that we describe below and tested the effect only during the last 3 s of the stream. The main goal of this follow-up study was to confirm the null result that we obtained in the primary study.

### Follow-up study

All experimental methods and analyses were preregistered here.

### Participants

In total, 37 unique participants, psychology students from the University of Groningen, were recruited and participated in the calibration and validation experiments for the follow-up study. From these, 30 participants (predetermined sample size) were eligible for participation in the main experiment upon signing the informed consent form. All participants had normal or corrected-to-normal vision. Participants received course credits for their participation. On the basis of a checklist developed by the Ethical committee (EC-BSS) at the University of Groningen, the study was exempt from full ethical review (PSY-2223-S-0165).

### Experimental design and procedure

The follow-up study was conducted in the same laboratory setup as the primary study. The follow-up study had the same structure as the primary study, containing calibration, validation, and main experiment. The calibration experiment was the same as in the primary study. However, the validation and the main experiment now featured only bright colors. The validation experiment consisted of 20 trials where both bright red and bright blue were presented 10 times in a randomized order. The main experiment consisted of 4 practice (not analyzed) trials and 200 experimental trials. Experimental trials were shuffled and divided in five blocks.

## Follow-up study: results

Data preprocessing was done as for the primary study. The same criteria for data exclusion were used as in the primary study. We excluded 901 trials in which the deviation from the center of the screen was larger than 6.93° (halfway to the letter stream) and lasted longer than 10 ms. We also excluded 675 trials containing baseline pupil sizes of ±2 *z*-scores that were considered outliers. In total, 1,576 trials (26.20%) were excluded from the data.

### Validation: directly looking at bright red/blue stimuli

To test the pupil size changes when directly looking at bright-red/blue stimuli, we ran the same analysis as in the primary study—LME.

**Fig. 4:**
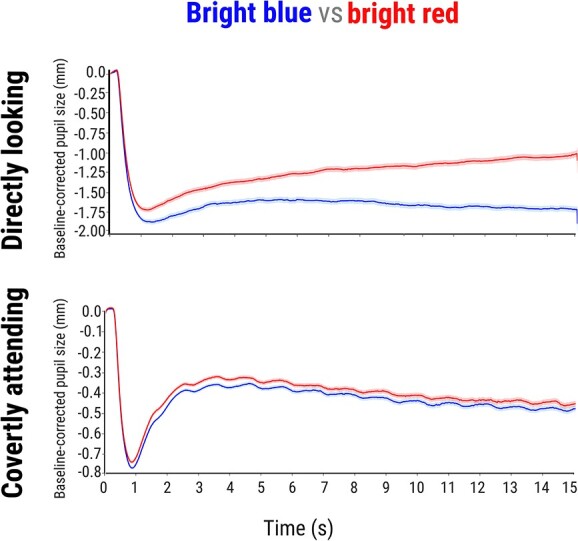
The pupillary results during direct gaze (the validation experiment; upper row) and covert attention (the main experiment; lower row) in the follow-up study. Pupil size when directly looking and covertly attending to bright blue vs. bright red. *Note:* Error bars represent the standard error.

We used the entire 15 s trace as a window of interest. Our model included mean pupil size as a dependent variable, color (red vs. blue) as a fixed effect, and by-participant random intercepts and slopes. We found a main effect of color (*b* = 0.40, *SE* = 0.03, *z* = 11.60, *P* < 0.001), reflecting that the pupil constricted more when directly looking at the blue trials as compared to the red trials (Fig. 4 upper row).

### Main experiment: covertly attending to bright red/blue stimuli

To test the pupil-size changes when covertly attending to bright-red/blue stimuli, we used the same analysis as in the primary study—combined cross-validation with LME. However, now we focused only on the last 3 s of the trace, which is a window of interest that we preregistered based on the exploratory results of the primary study (see above). We did not find a main effect of color (*z* = 0.63, *P* = 0.530, tested at samples 1,510, 1,350, and 1,580), suggesting that the there were no differences in pupil constriction when covertly attending to bright-red and bright blue stimuli (Fig. 4 lower row). That is, in a preregistered follow-up study that tested exactly the conditions under which the primary study showed a hint of an effect, we replicated our null result.

## Discussion

In our study, we investigated the effect of covert attention on the sustained phase of the PLR, which is mediated by ipRGCs; we compared this to the well-established effect of covert attention on the initial PLR (Binda et al. 2013; Mathôt et al. 2013; Naber et al. 2013), which is mediated by rods and cones. To do so, we exploited the fact that ipRGCs are most responsive to blue light, resulting in a prominent and sustained constriction when exposed to blue light stimuli. We found that the pupil constricted more when either directly looking at or covertly attending to bright as compared to dim stimuli (with the same color), replicating the effect of covert attention on the initial phase of the PLR. We also found that the pupil constricted more when directly looking at blue as compared to red stimuli (with the same luminosity); crucially, however, we did *not* find any difference in pupil size when covertly attending to blue as compared to red stimuli, which was also confirmed in a pre-registered follow-up study.

The effect of brightness (bright vs. dim) on the initial phase of the PLR, both when looking directly at a stimulus and when covertly attending to it, is illustrated in the left column of Fig. 2. Crucially, there is no effect of color on the initial phase of the PLR, neither when looking directly at a stimulus nor when covertly attending to it, as illustrated in the right column of Fig. 2; this is due to the fact that the we (successfully) controlled the influence of rods and cones by equating the luminance of the colors in order to isolate ipRGC activation during the sustained pupil response. During the direct looking condition, there was an effect of color (blue vs. red) on the sustained phase of the PLR; the activation of ipRGCs is slow, and as a result, this effect emerged only after approximately 5 s. As time progresses and ipRGCs become increasingly active, this difference becomes more pronounced, reaching approximately 0.4 mm toward the end (Fig. 2 right column, upper row).

The crucial finding of the present study is shown Fig. 2 right column, lower row. This shows that covertly attending to (unlike directly looking at) blue as compared to red does *not* result in increased sustained pupil constriction. This suggests that the sustained phase of the PLR, which is driven by ipRGCs, is impervious to covert attention. Interestingly, when we ran an exploratory analysis and looked at the effect of color on pupil size across different levels of brightness, we found a hint of an effect emerging in the last 3 s under the bright condition. Given that the activation threshold of ipRGCs typically requires photopic light levels exceeding 10 log quanta/cm^2^/s (Dacey et al. 2005), we ran a high-powered preregistered follow-up study focusing only on bright colors. However, we again did not find an effect, confirming that ipRGC-mediated pupil constriction is not susceptible to the effect of covert attention.

The fact that we observed a persistent pupil size difference when covertly attending to bright/dim stimuli may seem to conflict with the general idea, which we have also reiterated here, that rods and cones only contribute to the initial phase of the PLR and that sustained pupil constriction is driven by ipRGCs (Thompson et al. 2005; Park et al. 2011; Mathôt 2018; Lucas et al. 2020). If this is correct, then should we not observe that the effect of covertly attending to bright/dim stimuli is short-lived and disappears after several seconds as rods and cones adapt and ipRGCs take over? A likely explanation for the sustained effect that we observed here is that rods and cones did not undergo complete adaptation. Specifically, participants were continuously exposed to a stream of letters overlaid on static placeholders. With the presentation of each new letter, participants’ eyes naturally exhibited microsaccades. Both the letter changes and these microsaccades resulted in refreshing of rod and cone activation, especially because our placeholders had sharp edges that are continuously carried into and out of receptive fields. In other words, the sustained phase of the PLR could still be driven by rods and cones, which were repeatedly activated. Future research could test the effect of covert attention on ipRGCs while ensuring full adaptation of rods and cones. This could be accomplished using large and static stimuli without sharp edges, which would prevent microsaccades from reactivating rods and cones.

Moreover, future studies could aim for a more precise measurement of photoreceptor contribution, which we were not able to do with the current paradigm and apparatus. An effective method for achieving this precision is silent substitution, which involves carefully manipulating the spectral composition of light stimuli to selectively activate specific photoreceptor types while minimizing stimulation of others (Estévez and Spekreijse 1982; Zele et al. 2018; Nugent et al. 2023).

It is also important to consider the statistical power of our study in light of plausible effect sizes. Given that attention-induced effects on pupil size tend to be smaller in magnitude than light-induced effects, and considering that chromaticity (red vs. blue) contrast has a less pronounced impact on pupil responses compared to brightness (dim vs. bright) contrast under direct-looking conditions, one might wonder whether any difference in pupil size as a result of attending to red vs. blue is large enough to be detectable. In our investigation, we found that attention-induced effects were approximately four times smaller than light-induced effects when considering the dim vs. bright contrast (see also Binda et al. 2013). Based on this ratio, one might also expect the mean pupil size difference when attending to red vs. blue to be approximately four times smaller than the mean difference in pupil size when directly looking at red vs. blue, which was 0.19 mm. This would result in an anticipated difference of approximately 0.05 mm. However, the observed difference in pupil size when attending to red vs. blue was negligible (0.007 mm) in our study; combined with the fact that we conducted a high-powered study, this suggests that, at least in this paradigm, covert attention to red vs. blue really does not affect pupil size.

In conclusion, we replicated the effect of covert attention on the initial phase of the PLR, which is driven by rods and cones. However, across two experiments, we did not find any difference in pupil size when covertly attending to blue as compared to red stimuli (with the same luminosity), whereas we did observe this difference when participants directly looked at the same blue or red stimuli. This suggests that the sustained phase of the PLR, which is mainly driven by ipRGCs, is not modulated by covert attention. This is an important result since several studies have shown that ipRGC’s melanopsin-driven responses affect V1 activity as well as perceptual experiences (Spitschan et al. 2017; Allen et al. 2019), which are functions that are typically associated with attentional modulation. This finding also has important implications for our understanding of non-image-forming vision, that is, for visual functions that are not accompanied by visual awareness, such as regulation of circadian rhythm (Schmidt and Kofuji 2008; Zele et al. 2011). Since ipRGCs are strongly involved in non-image-forming vision (Güler et al. 2008; Schmidt and Kofuji 2008; Mathôt 2018), our results imply that the pathway that underlies non-image-forming vision may—unlike image-forming vision—not be modulated by covert visual attention.

### Open-practices statement

The preregistration, experimental data, the experiment, and analysis scripts related to the primary study can be found here.

The preregistration, experimental data, the experiment, and analysis scripts related to the follow-up study can be found here.

## Author contributions

Ana Vilotijević (Conceptualization, Data curation, Formal analysis, Investigation, Methodology, Project administration, Visualization, Writing—original draft, Writing—review & editing, Validation) and Sebastiaan Mathôt (Conceptualization, Data curation, Methodology, Funding acquisition, Software, Supervision, Writing—review & editing, Validation).

## Funding

This research was supported by the Innovational Research Incentives Scheme VIDI (VI.Vidi.191.045) from the Dutch Research Council (NWO) to S.M.

##  

*Conflict of interest statement*: None declared.

## Supplementary Material

Appendix_Vilotijevic_Mathot_2024_bhae107
