# Supplementary material for: Non-image-forming vision as measured through ipRGC-mediated pupil constriction is not modulated by covert visual attention
Source: Cereb Cortex. 2024 Mar 23;34(3):bhae107. doi: 10.1093/cercor/bhae107 (PMC10960954; doi:10.1093/cercor/bhae107)
Supplement: Appendix_Vilotijevic_Mathot_2024_bhae107 [file appendix_vilotijevic_mathot_2024_bhae107.docx]

# **Appendix**


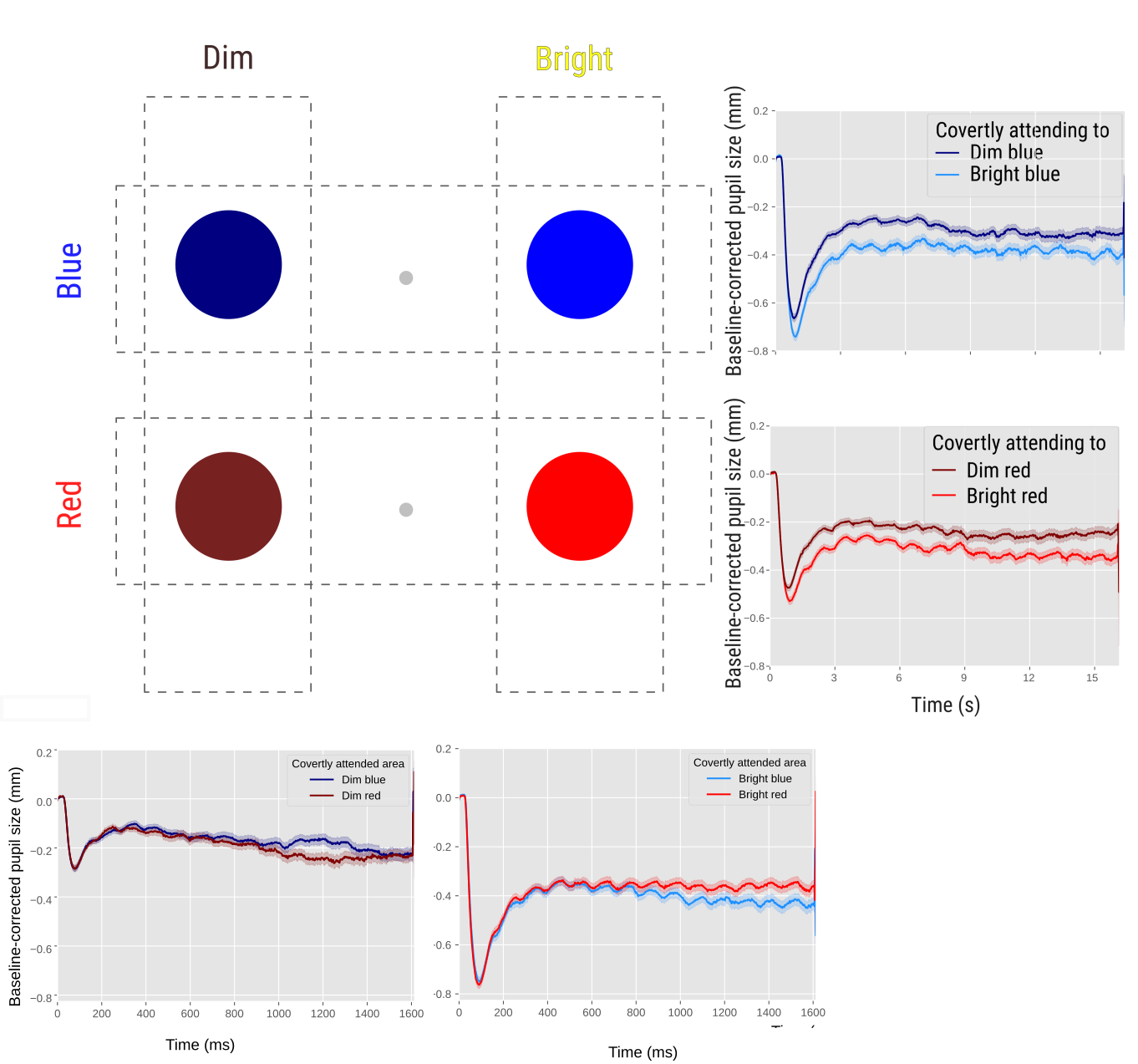


*Figure 5.* Separate comparisons from the primary study. Pupil size when covertly attending to bright/ dim blue/ red under the same color (rows)/ brightness (columns) conditions.

# *Omnibus test (Exploratory analysis)*

As an additional exploratory analysis, we ran the cross-validation in combination with LME on the combined data from both primary and follow-up studies (*N* = 60). More specifically, we selected only bright trials from the primary study and merged those to the follow-up study. We found no effect of color (bright red vs bright blue) on pupil size when the last three seconds of the stream were analyzed (*z* = 1.67, *p* = .09, tested at samples 1430, 1420, 1570, and 1340), suggesting that there were no differences in pupil constriction when covertly attending to blue as compared to red placeholders. Surprisingly, upon the analysis of the complete 15-second trace, the effect turned out to be statistically significant (*z* = 2.69, *p* = .007, tested at samples 170, 1610, 160). However, it is crucial to note that most of the samples identified through cross-validation pertain to activity within the initial two seconds following stimulus onset, which is inconsistent with the typical latency of ipRGCs response. Most likely, there were slight imperfections in the brightness calibration of red and blue intensities, resulting in a sustained difference in pupil size already from the onset of the pupil response; this difference is numerically very small, but because of the very large sample size included in this omnibus analysis, it was statistically significant (for more details see omnibus-test-notebook [here](https://osf.io/q2d63/)).
